# Cooperation and conflict in intra‐hospital transfers: A qualitative analysis

**DOI:** 10.1002/nop2.434

**Published:** 2019-12-17

**Authors:** Hayley D. Germack, Renee Fekieta, Meredith Campbell Britton, Shelli L. Feder, Alana Rosenberg, Sarwat I. Chaudhry

**Affiliations:** ^1^ National Clinician Scholars Program Yale University School of Medicine New Haven CT USA; ^2^ Department of Population Health Yale University School of Medicine New Haven CT USA; ^3^ Equity Research and Innovation Center Yale University School of Medicine New Haven CT USA; ^4^ Yale University School of Nursing New Haven CT USA; ^5^ School of Public Health Yale University New Haven CT USA; ^6^ Department of Internal Medicine Yale University School of Medicine New Haven CT USA; ^7^ Present address: School of Nursing Department of Acute and Tertiary Care University of Pittsburgh Pittsburgh PA USA

**Keywords:** care transfers, intra‐hospital transfers, multidisciplinary, qualitative study, quality improvement

## Abstract

**Aim:**

The purpose of this study was to explore the latent conditions of cooperation and conflict in intra‐hospital patient transfers (i.e. transfers of patients between units in a hospital).

**Design:**

Secondary qualitative analysis of 28 interviews conducted with 29 hospital staff, including physicians (*N* = 13), nurses (*N* = 10) and support staff (*N* = 6) from a single, large academic tertiary hospital in the Northeastern United States.

**Methods:**

A two‐member multidisciplinary team applied a directed content analysis approach to data collected from semi‐structured interviews.

**Results:**

Three recurrent themes were generated: (a) patient flow policies created imbalances of power; (b) relationships were helpful to facilitate safe transfers; and (c) method of admission order communication was a source of disagreement. Hospital quality improvement efforts could benefit from a teaming approach to minimize unintentional power imbalances and optimize communicative relationships between units.

## 1 INTRODUCTION

Intra‐hospital patient transfers (i.e. transfers of patients between units in a hospital) are common and pose risks to patient safety. Thousands of transfers between hospital emergency departments, inpatient floors and intensive care units occur in USA hospitals each day (Rui, Kang, & Albert, 2013). Transfers introduce risk for adverse events, including errors of diagnosis, treatment (Bell, Rahimi‐Darabad, & Orner, 2006) and disposition (processes involved in determining the right floor and bed for a particular patient; Horwitz et al., 2009). Regulatory agencies, accrediting organizations, healthcare administrators and healthcare providers are committed to mitigating the numerous threats that intra‐hospital transfers pose to patient safety (Joint Commission, 2017a; Kohn, Corrigan, & Donaldson, 2000; Snow et al., 2009).

### 1.1 Background

Intra‐hospital transfers are challenging because they demand that large quantities of patient information are accurately and efficiently conveyed between the sending and receiving units. In fact, in 2009, the Joint Commission Center for Transforming Healthcare (2018; a group of leading hospitals and health systems in the United States) identified improving transfer communication between individuals in different care settings as a targeted initiative. There is a growing body of literature on transfers between providers on different units in a single hospital (Cohen & Hilligoss, 2010; Detsky, Ailon, Weinerman, Amaral, & Bell, 2015; Hilligoss & Cohen, 2013; Hilligoss, Mansfield, Patterson, & Moffatt‐Bruce, 2015; Lyons, Arora, & Farnon, 2016; Ong & Coiera, 2011). Much of this literature is limited to transfers of care between providers of the same profession (i.e. nurse‐to‐nurse or physician‐to‐physician) (Detsky et al., 2015; Li, Stelfox, & Ghali, 2011; Lyons et al., 2016). The outcomes examined in these studies included clinical adverse events (Lyons et al., 2016; Marquet et al., 2015; Starmer et al., 2013) and delays in care (Sankey, McAvay, Siner, Barsky, & Chaudhry, 2016).

While it is critical to understand the risks posed to patients during transfers, the impact of transfers on hospital staff from a wide array of professional backgrounds is less well‐understood (Halvorson et al., 2016; Rosenberg et al., 2018). Research that acknowledges staff experiences in maintaining patient safety during intra‐hospital transfers is needed to understand other factors that contribute to adverse events, delays in care and other risks to patients (Hearld, Alexander, Fraser, & Jiang, 2008). To this end, a quality improvement project using an ethnographic approach was conducted to examine the latent conditions that affected how multidisciplinary team members experienced intra‐hospital transfers from the Emergency Department and Medical Intensive Care Unit to General Internal Medicine floors at an urban teaching hospital (Rosenberg et al., 2018). Observations of and interviews with team members including clinicians (nurses, physicians and a physician's assistant) and support staff (unit clerks and bed managers and bed associates who assigned patients to beds) informed the development of a taxonomy of intra‐hospital transfers (Rosenberg et al., 2018). Cooperation and conflict were two prominent codes that emerged from the primary analysis, leading the research team to suggest further study.

**Table 1:** Illustrative quotes across the three themes

Patient flow policies created imbalances of power	
Unit transfer privilege	*The* *emergency department … just [books] them to medicine … they don't call you to say, “Can I book them to medicine?” They just say, “We booked a patient to you.”*Receiving general medicine physician on unit transfer privilege policy
	*That's been a problem…that the patients don't go to the right services, because they [the emergency department] would call urology and urology would say, “No, I know the patient has kidney stones and kidney failure, but [he] also has bad COPD [chronic obstructive pulmonary disease], so book him to medicine, and we'll just consult.”—*Receiving general medicine physician on unit transfer privilege
	*It's pretty smooth sailing…there's really no resistance or push back from the floor…it's pretty straightforward…they don't have the right to refuse patients.—*Sending emergency department physician on unit transfer privilege
Four‐hour mark	*There's a lot of pushing and shoving on both ends. The emergency department will call at the 3 hr and 59 min mark. When we're really busy in the emergency department, and there are no beds, they are assigned to a hospitalist service. We have four hours. At 3 hr and 50 min, we get calls, “You know you have to assign that?”*—Bed management on four‐hour mark
	*What we found is that we'd get a call at that four‐hour mark, but we would find out that the patient has had nothing ordered or no evaluation for hours before that. They would call you with, say, “By the way, it's been four hours, and the patient is having these symptoms and needs you right now.”*—Receiving hospitalist physician on four‐hour mark
Relationships	*We don't necessarily have the working relationship that we do with the nurses on the floor, so it's harder*.—Receiving general medicine physician
	*The implementation is obviously an issue. You have people going to these meetings, and they agree on things, but when it comes to implementing…*—Sending emergency physician
	*Resources and relationships, obviously that's a big, huge part of transitions of care that fixes it, relationships.*—Sending emergency physician
	*I think having open communication through this emergency department/medicine huddle helps*—Sending emergency physician
	*It's nice to know that there's attention to it and both parties are trying to work on it—because sometimes you feel like you're the only one working on the problem.*—Receiving nurse
	*They should get to be in the other person's shoes. Do some time up in the intensive care unit. They come down to the emergency department for even if it's like half a day, just to see what it's like. You have that perspective, because when we're calling you to report on the patient, I know you have another sick, sick patient, you really need 15 min, but I may have four intubated patients that need to go to the intensive care unit. You're the only bed open, and I need to relieve one of my problem children to you. Having that understanding that we don't have the ability to stop.*—Sending emergency nurse
Admission order communication	
Conflict over calling versus. using the EHR	*I personally feel that the information is in [the electronic medical record], and if we're doing a good job of documenting the patient's condition within our documentation, they [the receiving unit] should see that. We should not need to put another barrier of a phone call in there.*–Sending nurse
	*It was just nice to have that little heads‐up. Plus you're talking to another nurse and she can just—you can get a feeling of what's coming your way—*Receiving nurse
Scope of practice policies	*The other thing is the nursing rules or stuff like that. We're not always clear about those. I think it would be helpful to know what actually is allowed on each floor and what's acceptable, what's not acceptable.*—Sending physician
	*“Can you come find the Scope of Practice Policy, because I need to show this doctor that we don't do it?” They don't necessarily trust the word of mouth...—*Receiving nurse
	*I think at the end of the day if they're receiving and this is not comfortable, usually from a nursing perspective, then we just—we get stuck and we kind of hold onto the patient.—*Sending physician

Note

Illustrative quotes are presented according to the conventions for presenting verbatim quotations of participants in qualitative and social research (Corden & Sainsbury, 2006). Quotes were italicized to indicate the participant's own words and followed by a general label to identify the speaker while still ensuring anonymity. Quotes were presented as close to verbatim with as little editing as possible. Ellipses (*…*) were inserted to indicate places where words or phrases were omitted (such as verbal hesitations to enhance readability and avoid the repetition and false starts heard in conversational speech). General or explanatory terms were placed within square brackets [ ].

The purpose of this secondary qualitative analysis was to build on findings from a quality improvement project of hospital staff experiences with intra‐hospital patient transfers by exploring the latent conditions of cooperation and conflict more deeply. A directed content analysis of data coded for cooperation and conflict was conducted to generate themes about how staff from multiple disciplines experience and view cooperation and conflict during intra‐hospital patient transfers (Hsieh & Shannon, 2005).

## 2 METHODS

The following methods and results are reported in accordance with the Consolidated Criteria for Reporting Qualitative Research (COREQ; Tong, Sainsbury, & Craig, 2007).

### 2.1 Design

The quality improvement project—herein referred to as the parent study—intended to: (a) better understand the process of transferring patients from the emergency department and medical intensive care unit to the general internal medicine floors of an urban teaching hospital; (b) identify challenges and opportunities for improvement in the transition process; and (c) develop interventions to reduce adverse patient events as well as increase staff satisfaction with the transition process. Details of the parent study including the sampling process, data collection (i.e. where the interviews were conducted, who transcribed, how long the interviews were) and data analysis, are well‐described in the parent publication (Rosenberg et al., 2018). The parent study identified sending units (units that send patients) and receiving units (units that receive patients) with the greatest number of transfers and staff complaints related to transfers. Participants (*N* = 29) from the sending and receiving units consisted of 13 physicians (including a unit medical director and an associate medical director), 10 nurses (including staff nurses, a patient services manager and a nurse manager) and six support staff (including a clinical bed manager and a bed associate). The parent study analysis team was surprised by the frequency of cooperation and conflict codes—not only did the codes occur often, but these two seemingly contradictory codes occurred frequently together. Twenty‐seven of the original 28 interview transcripts included both the cooperation and conflict codes. The team felt that the prominence of these codes warranted further analysis to understand how cooperation and conflict were experienced during care transfers.

### 2.2 Sampling

The secondary analysis team had access to the full transcribed and coded interviews in ATLAS.ti (2017) qualitative software, version 7.0 (Scientific Software). The secondary analysis sampled interview data coded with the cooperation and/or conflict codes.

### 2.3 Analysis

The secondary analysis occurred between February and November 2017. The secondary analysis of the cooperation and conflict codes took place in Atlas.ti. Two researchers (disciplines of nursing‐and‐health services research and quality improvement) immersed themselves in the interview data coded with conflict and cooperation (Patton, 2015). The two‐member team was not part of the original coding team, but had access to the data through a formal research relationship with the project's principal investigator (Heaton, 2004). Both members of the team had prior qualitative research experience including coding and analysis. The researchers used directed content analysis, whereby they analysed data coded for cooperation and conflict in the parent study and synthesized the qualitative data to identify patterns and develop themes across experiences (Hsieh & Shannon, 2005). A new code structure was created for this analysis. The researchers performed line‐by‐line reviews and assigned open codes first working independently, then together. The team met regularly to discuss and refine definitions for open codes, continuously updated the coding structure to reflect the emerging data, refine code definitions and organize findings. Once coding of the data set was complete, the team analysed code reports and developed themes.

Analytic rigour was supported through coding procedures, including maintaining an audit trail through memos kept in Atlas.ti to document the analytic process. The trustworthiness of data analysis was established by reviewing themes in a series of feedback sessions between the secondary analysis team and the parent study team (Creswell & Miller, 2000). During these sessions, the study team shared themes that emerged from the secondary analysis and incorporated insights from the parent study team to deepen interpretation. Disagreements were resolved through discussion with the parent study's three‐member research team until consensus was reached. Final code structure was reviewed with the study's PI’s in two meetings with the secondary analysis study team.

### 2.4 Ethical considerations

The Institutional Review Board (IRB) at the senior author's institution reviewed and exempted the parent study as quality improvement. This secondary analysis of the parent study was permitted as the institution's IRB has published guidelines permitting the publication of works related to quality improvement initiatives. Participants were asked for verbal consent to conduct and record the interview before it began. The consent process included a discussion about what types of information may be collected and analysed, including interpersonal communication related to the in‐hospital transfer process.

## 3 RESULTS

**Figure 1:**
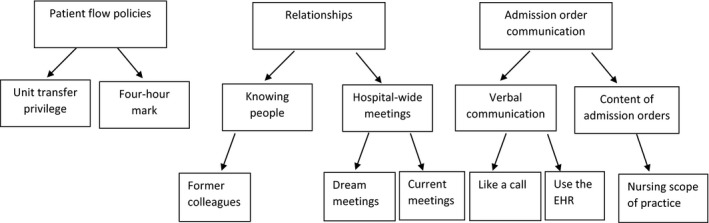
Thematic tree

The research team identified three themes in the data: (a) patient flow policies created imbalances of power; (b) relationships were helpful to facilitate safe transfers; and (c) method of admission order communication was a source of disagreement (Figure 1). Illustrative quotes are displayed in Table 1.

### 3.1 Patient flow policies

Senders, receivers and support staff identified several hospital policies that complicated the transition process by creating imbalances of power and conflict among groups. These patient flow policies epitomize the struggle of working within the mandates of a complex system that staff perceived as sometimes impeding high‐quality and safe care transfers.

At the time of the parent study, there were two specific hospital policies in place designed to improve patient flow and reduce crowding in the emergency department: the unit transfer privilege and the four‐hour mark. Both policies focused on the transfer process between the emergency department and general medicine service. Both policies were aimed at decreasing length of stay in the emergency department. The four‐hour mark is a controversial performance indicator that stipulates that all patients presenting to emergency departments should be seen and treated or admitted to a hospital bed within 4 hr of arriving (Higginson, Kehoe, Whyatt, & Smith, 2017).

Under the unit transfer privilege policy, emergency physicians had the authority to book patients directly to the general medical service without consulting receiving providers. The general internal medicine unit was the only unit in the hospital that the emergency department could book to directly, without consulting with the receiving providers for acceptance. Other specialty units, such as urology or oncology, required receiving provider approval. This unit transfer privilege was characterized as helpful by sending care teams and disempowering by receiving care teams. Specifically, one emergency physician described the policy as marked by a high degree of cooperation and “smooth sailing.” Receiving staff, on the other hand, emphasized the disempowering effects of this policy. A general medicine physician juxtaposed his perception of the policy—as a passive receiver awaiting patients on his unit—to those providers who had an active role in the transfer process (Table 1). The same general medicine physician described how this policy had a negative impact on patient disposition. The general medicine physician's perception was that this patient would be more appropriately placed on a specialty unit where he could be treated for his primary presenting problem, namely kidney stones and kidney failure. The same policy, which the emergency department experienced as cooperative, generated conflict when imposed on the general medical staff.

Another contested policy was the four‐hour mark, wherein the responsibility of patient care was automatically transferred from the emergency department physician to a general medicine physician after the patient had been in the emergency department for 4 hr. This policy was implemented in response to a Joint Commission standard for hospital accreditation (Joint Commission, 2011, 2013). Staff from the bed management department, who placed patients throughout the hospital, often used those 4 hr to assign patients in the hopes that a bed would free up on a medical unit and the patient could be physically transferred out of the emergency department. This would prevent a general medicine physician from having to travel to the emergency department to provide clinical care. However, by waiting to assign the patient, the bed manager limited the amount of notice that the general medicine physician received in preparation to care for a patient in a different part of the building (i.e. the emergency department vs. the general medicine ward). Intended to expedite flow, this policy left the receiving providers, who were not physically present on the same unit as the patients they were caring for, feeling frustrated about their ability to provide safe care. The same policy that sending providers viewed as facilitating transfers frustrated the receiving providers.

### 3.2 Relationships

Participants described their relationships with staff on other units as helpful to facilitating safe patient transfers. These relationships could include previous experience working on another unit, membership or participation in hospital‐wide committees and events, or even friendships with staff members from other departments. Participants reflected that often simply knowing the “right” people across units could help make a transfer successful. A sending emergency physician, for example, discussed how transfers go more smoothly for her because of her history at the hospital. Conversely, a receiving general medicine physician described challenges when caring for patients in the emergency department, where he does not have familiarity with staff.

Staff also discussed opportunities for enhanced cooperation that could strengthen relationships between units. A sending emergency nurse, for example, imagined a programme during new nurse orientation, which introduces them to the hospital—highlighting the benefits of shadowing other teams. Other participants shared their experiences with hospital‐wide quality improvement meetings, where staff members from different teams work together to identify potential solutions to patient safety challenges. A sending physician and a receiving nurse appreciated the dialogue these meetings created. However, a receiving physician lamented about the lack of concrete follow‐up activity (Table 1).

### 3.3 Admission order communication

Admission orders are essential to any intra‐hospital transfer. They serve to inform the beds management team and receiving unit about what care a patient will need, influencing appropriate disposition. At the time of data collection, there had been a recent (within the past 2 years) change in admission order communication—an electronic medical record‐based checklist had replaced the verbal component of transfers between emergency and general medicine nurses. This change was a frequent source of debate among participants. When valuable information that influenced a patient's placement and, thus, the receiving unit's ability to care for patient was left out of an admission order, conflict ensued.

Participants espoused two schools of thought on the necessity of verbal transfer communication in addition to the electronic medical record‐based checklist. Staff who had been at the hospital for a long time remembered the pre‐electronic medical record days. The electronic medical record‐based checklist was intended to be a streamlined communication mechanism, replacing the need for face‐to‐face or phone communication between providers. However, some clinicians valued the extra verbal exchange with a receiving provider and saw these conversations as valuable to their safe preparation for a new patient.

Other staff felt that the checklist replaced the need for face‐to‐face transfer communication between providers. A sending nurse, on the other hand, considered verbal exchange of information as unnecessary and a waste of time (Table 1). This nurse did not value the mode of communication that providers from other units found critical to safe transfers.

In addition to mode of communication being a source of disagreement, the content of admission orders hindered transfers. Nursing scope of practice policies—policies that dictate what nurses can and cannot do—varied across units. Sending providers’ lack of knowledge about variation in policies contributed to transition inefficiencies and were marked by more conflict than cooperation. Sending providers were sometimes unaware of limitations in receiving units’ scope of practice and therefore did not always include pertinent patient care in the admission orders. Receivers described receiving patients that they were unable to treat, such as patients requiring additional telemetry monitoring for specific cardiac medications. Often, this resulted in a re‐booking of the patient to a different unit. Occasionally, scope of practice policy was interpreted as lack of confidence in caring for specific patients. While a sending physician interpreted units not accepting patients as within the control of the receiving nurses, a receiving nurse described having to serve as an arbiter for the frequent conflicts that arose when sending teams were unaware of these unit‐specific policies and did not understand limitations in scopes of practice.

## 4 DISCUSSION

This study sought to understand and characterize the multidisciplinary staff experience of cooperation and conflict during intra‐hospital transfers. The directed content analysis generated three themes: (a) patient flow policies created imbalances of power; (b) relationships were helpful to facilitate safe transfers; and (c) method of admission order communication was a source of disagreement.

Sending and receiving staff were asked to adhere to transition policies that facilitated transfers for some providers, but left others feeling disempowered. Despite having opportunities to voice concerns in formal venues including huddles and safety reporting, conflict was simmering at the staff level rather than moving up to administration. The mechanisms that allowed staff to file complaints with the administrators were indeed functioning. Relationships with staff on other units helped them navigate the myriad challenges of intra‐hospital transfers, but not having these relationships complicated the transfers for some clinicians. Staff also acknowledged the importance of sharing information about patients but reported that incomplete communication frequently occurred. Specifically, lack of patient information in admission orders and lack of senders’ awareness of receiving unit nurse scope of practice policies complicated appropriate patient disposition. These findings have important implications for the development of interventions to improve intra‐hospital care transfers.

This study—along with the parent study—builds on the existing literature that had focused on transfers and communication between only‐physicians (Detsky et al., 2015; Hilligoss et al., 2015) or between only‐nurses (Shields, Overstreet, & Krau, 2015) by incorporating diverse stakeholder perspectives from across the transfer process. This study continues to expand the scope of intra‐hospital transfer literature, which has narrowly focused on failures in communication (Ong et al., 2011; Patterson & Wears, 2010), by considering the roles of other factors, including patient flow policies and relationships. Our results are aligned with other studies that have highlighted gaps in communication, relationships and process. In a two‐site survey of intensive care unit and general internal medicine physicians focusing on intra‐hospital transfers, Detsky et al. (2015) found that both sending and receiving physicians agreed that the existing process for transfer of information (which consisted mostly of written chart notes and telephone communication, but almost a third of the time, no verbal or written communication) could be improved and contributed to adverse events, including medication errors, aspiration and mental status decompensation requiring response. Notably, the authors identified the inclusion of other health care workers as an important step for future work in this area (Detsky et al., 2015). Hilligoss et al. (2015) characterized the challenges of between‐unit transfers after a 2‐year ethnographic study of intra‐hospital transfers between emergency and intensive care unit and general medicine physicians at two different medical centres. Those findings include unequal distributions of power among units, infrequent face‐to‐face communication and a lack of awareness of the other unit's state (Hilligoss et al., 2015). However, both studies were restricted to communication of information during transfers and limited to the perspectives of physicians.

This study expands the literature by capturing the voices of multiple staff involved in transfers and applying a systems approach that highlights the impact of hospital policy within a broader social context (Alexander & Hearld, 2012). Our analysis was novel because we characterized the moments when participants felt most frustrated, or most supported and related these characterizations directly to policies and structural barriers at the hospital. Our analysis highlights the importance of examining organizational‐wide policies and unit‐specific cultural adaptations to those policies. The study also demonstrated that conflict is not necessarily terminal‐conflict can be an indicator of a problem or potential risk. In line with literature on group dynamics, conflict is essential to healthy group functioning (Smith & Berg, 1987). Our findings also revealed that cooperation in the transition process can belie the disempowerment experienced by participants as evidenced by the experiences of bed management with the four‐hour mark.

We used established approaches (e.g. a multi‐member coding team, an audit trail using memos and feedback sessions) to enhance the rigour of our findings (Miles, Huberman, & Saldaña, 2014; Patton, 2015); however, our study is not without limitations. First, our findings cannot be generalized to all hospitals. However, qualitative study findings can provide insights into areas that have been previously unexplored and can generate hypotheses for future quantitative evaluations (Curry, Nembhard, & Bradley, 2009). Second, as this study was based on interview data, we must acknowledge the potential impact of social desirability bias (Collins, Shattell, & Thomas, 2005)—participants may have misrepresented their role in transfers to provide desirable answers. Third, as a secondary analysis of qualitative data, the data were limited to what was available in the parent study (Hinds, Vogel, & Clarke‐Steffen, 1997).

## 5 CONCLUSION

Our findings have implications for improving the day‐to‐day experience of staff navigating transfers of patients between units. In a report by the Joint Commission Center for Transforming Healthcare (Joint Commission, 2017b), a lack of teamwork and respect was identified as a specific root cause for failures in care transfers. Two of their proposed solutions addressed the culture of transfers and encouraged organizations to make successful transfers an organizational priority. Institutional use of a standardized transfer tool developed by all members of the intra‐hospital transition staff in a mutually agreed on medium (e.g. verbal, written or in the chart) could foster a culture of shared responsibility while standardizing critical content for efficient communication. Recent work by Abraham and Acharya (2016) applied the theory of “common information spaces” to develop evidence‐based guidelines for an interdisciplinary handoff framework and embraced the similarities between resident physicians’ and nurses’ transfer communication. Applying a teaming approach, which includes representation from all involved departments, could minimize unintended consequences of hospital‐wide policies. Quality improvement efforts spanning units, with goal‐setting that crosses boundaries and optimizes synergies between units, could be an efficient route for solidifying these relationships. Hospital policies should be examined from the perspective of multiple units. Rather than seek to eliminate all conflict, which is likely impossible, future quality improvement efforts should consider how to learn from experiences of conflict as indicators of staff burnout or potential risks to patients.

Hospital staff involved in intra‐hospital transfers must navigate a complex healthcare system to get patients to the appropriate site of care. Nurses, physicians and support staff described episodes of cooperation and conflict—some complicated by hospital policy—pervading the relationships and communication that occurred between units. The emergence of these domains across different types of staff underscores the importance of integrating the whole healthcare team in organization‐wide problems and solutions.

## CONFLICT OF INTEREST

The authors declare no conflicts of interest to disclose.
